# Genetic basis for retention of a critical virulence plasmid of *Borrelia burgdorferi*

**DOI:** 10.1111/j.1365-2958.2007.05969.x

**Published:** 2007-11-05

**Authors:** Mollie W Jewett, Rebecca Byram, Aaron Bestor, Kit Tilly, Kevin Lawrence, Mary N Burtnick, Frank Gherardini, Patricia A Rosa

**Affiliations:** 1 Laboratory of Zoonotic Pathogens, Rocky Mountain Laboratories, National Institute of Allergy and Infectious Diseases, National Institutes of Health Hamilton, MT 59840, USA.; 2 Department of Molecular Biology, University of Wyoming Laramie, WY 82701, USA.

## Abstract

The genome of *Borrelia burgdorferi* is composed of one linear chromosome and approximately 20 linear and circular plasmids. Although some plasmids are required by *B. burgdorferi in vivo*, most plasmids are dispensable for growth *in vitro*. However, circular plasmid (cp) 26 is present in all natural isolates and has never been lost during *in vitro* growth. This plasmid carries *ospC*, which is critical for mammalian infection. We previously showed that cp26 encodes essential functions, including the telomere resolvase, ResT, and hence cannot be displaced. Here we identify two additional essential genes on cp26, *bbb26* and *bbb27*, through a systematic attempt to inactivate each open reading frame (ORF). Furthermore, an incompatible plasmid carrying *resT*, *bbb26* and *bbb27* could displace cp26. Computational and experimental analyses suggested that both BBB26 and BBB27 are membrane-associated, periplasmic proteins. These data indicate that *bbb26* and *bbb27* encode essential but possibly redundant functions and that one or the other of these cp26 genes, in addition to *resT*, is required for bacterial viability. We conclude that the genetic linkage of critical physiological and virulence functions on cp26 is pertinent to its stable maintenance throughout the evolution of *B. burgdorferi*.

## Introduction

*Borrelia burgdorferi*, the spirochetal agent of Lyme disease, is maintained in nature in a complex cycle between small mammals and a tick vector of the genus *Ixodes*. The genome of *B. burgdorferi* type strain B31 has been sequenced and consists of an approximately 900 kbp linear chromosome and more than 20 plasmids that together total ∼600 kbp in size (Stevenson *et al*., 1996; 1997; Fraser *et al*., 1997; Casjens *et al*., 2000; Miller *et al*., 2000). The 21 plasmids present in the sequenced *B. burgdorferi* B31 strain include 12 linear and nine circular replicons (Fraser *et al*., 1997; Casjens *et al*., 2000). Additional circular plasmids have been described in other stocks of strain B31, suggesting that at least 23 different plasmids were present in the original B31 isolate (Stevenson *et al*., 1996; 1997; Casjens *et al*., 2000; Miller *et al*., 2000). There is increasing evidence to suggest that plasmid-derived functions can be critical for *B. burgdorferi* viability at various stages in the natural mouse-tick infectious cycle (Purser and Norris, 2000; Labandeira-Rey and Skare, 2001; Purser *et al*., 2003; Grimm *et al*., 2004a; 2005; Pal *et al*., 2004; Yang *et al*., 2004; Revel *et al*., 2005; Strother *et al*., 2005; Jewett *et al*., 2007). For example, linear plasmids of 25 kbp (lp25), 28 kbp (lp28-1) and 36 kbp (lp36) are important for infectivity in the mouse (Purser and Norris, 2000; Labandeira-Rey and Skare, 2001; Purser *et al*., 2003; Jewett *et al*., 2007) and lp25 is also required for survival in the tick (Grimm *et al*., 2005; Revel *et al*., 2005; Strother *et al*., 2005). Although plasmid-encoded proteins may be required for *B. burgdorferi* survival *in vivo*, plasmid loss can be observed after limited *in vitro* propagation (Xu *et al*., 1996; Schwan *et al*., 1988; Grimm *et al*., 2003). In fact, serial passage coupled with immune selection against plasmid-encoded surface proteins yielded the *B. burgdorferi* clone B314 that lacks all linear and most circular plasmids (Sadziene *et al*., 1993). However, the loss of one circular plasmid of 26 kbp (cp26) has never been observed and this plasmid is present in all isolates that have been examined (Marconi *et al*., 1993; Tilly *et al*., 1998; Casjens *et al*., 2000; Byram *et al*., 2004; Terekhova *et al*., 2006). Furthermore, in contrast to other *B. burgdorferi* plasmids, cp26 cannot be displaced by an incompatible plasmid harbouring the cp26 replication machinery alone, suggesting that this plasmid carries essential genes (Byram *et al*., 2004).

One cp26 gene that appears to be critical for bacterial viability is *resT*, which is present as a single copy and encodes an enzyme required for resolution of the replicated telomeres of the linear DNA molecules, including the chromosome, into covalently closed hairpin ends (Kobryn and Chaconas, 2002; Byram *et al*., 2004; Chaconas, 2005). The *resT* gene could not be inactivated by allelic exchange unless a functional homologue was also present (Tourand *et al*., 2006) and is presumed to be required for spirochete growth (Byram *et al*., 2004). However, an incompatible plasmid that carried *resT* was unable to displace cp26, suggesting that telomere resolution is not the sole essential function encoded by cp26 (Byram *et al*., 2004).

**Fig. 1:**
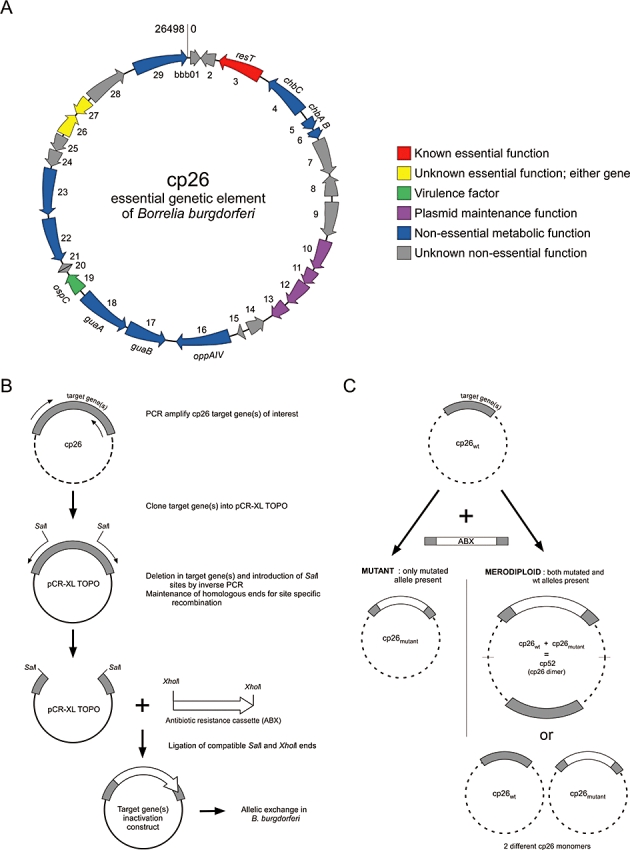
A. Graphical representation of cp26. The arbitrary position of bases 0/26498 is represented by a vertical line between *bbb01* and *bbb29* (Fraser *et al*., 1997). Genes are grouped according to functional class. Known essential function (red); unknown essential function (yellow); virulence factor (green); plasmid maintenance (purple); non-essential metabolic function (blue); unknown non-essential function (grey). B. Schematic diagram of the method used for targeted mutagenesis of most of the genes on cp26. Targeted mutagenesis was achieved by allelic exchange of an antibiotic resistance cassette with the gene(s) of interest. C. Graphical representation of the possible outcomes of allelic exchange with cp26 target genes. A single mutated copy of a gene(s) can be recovered if the gene(s) is non-essential for growth. Merodiploids are defined as clones that harbour both a wild-type and mutant copy of the target gene(s) within the same cell, in this case either on a single cp26 dimer (cp52) or on two separate, coexisting cp26 monomers.

Twenty-nine ORFs are present on cp26 and approximately half of these genes have known or putative functions (Fig. 1A) (Fraser *et al*., 1997; Casjens *et al*., 2000). Along with *resT*, cp26 carries genes that have been proposed or shown to be involved in purine biosynthesis (*guaA* and *guaB*) (Margolis *et al*., 1994; Zhou *et al*., 1997), peptide transport (*oppAIV*) (Bono *et al*., 1998), chitobiose transport (*chbA*, *chbB* and *chbC*) (Tilly *et al*., 2001), glucose transport (*bbb29*) (Fraser *et al*., 1997; Casjens *et al*., 2000; Byram *et al*., 2004) and purine transport (*bbb22* and *bbb23*) (Byram *et al*., 2004). In addition, the cp26-encoded outer surface lipoprotein C (OspC) is required for spirochete survival in the mammalian host (Grimm *et al*., 2004a; Stewart *et al*., 2006; Tilly *et al*., 2006; 2007). Although some of the above-mentioned cp26 genes encode products that are known to be important to *B. burgdorferi* for growth and maintenance in its natural infectious cycle, *ospC* (Tilly *et al*., 1997), *chbC* (Tilly *et al*., 2001), *oppAIV* (Bono *et al*., 1998), *guaB* (Tilly *et al*., 1998) and *bbb29* (Byram *et al*., 2004) have all been inactivated in previous studies without altering the spirochete's ability to propagate under normal *in vitro* growth conditions.

In this study, we undertook to inactivate all remaining cp26 ORFs to determine which functions encoded by this plasmid, in addition to telomere resolution, are required for *B. burgdorferi* growth *in vitro*. We then attempted to selectively displace cp26 using an incompatible plasmid that carried, along with *resT*, those genes implicated to be important for bacterial survival. Our findings identify the complement of genes on cp26 that ensures stable retention of this replicon by *B. burgdorferi* throughout its life cycle.

## Results

### Inactivation of all cp26 ORFs except the essential *resT* gene and those required for plasmid maintenance

In a previous study we concluded that cp26 encodes functions essential to bacterial viability because of its presence in all natural isolates, its stability during *in vitro* growth, and its inability to be displaced by an incompatible plasmid harbouring only the cp26 replication machinery (Byram *et al*., 2004). We also discovered that telomere resolution, encoded by the cp26 gene *resT*, is a function vital for *B. burgdorferi* survival (Byram *et al*., 2004). However, transformation of *B. burgdorferi* with a cp26-incompatible plasmid carrying the essential *resT* gene did not result in displacement of cp26, suggesting that additional genes on cp26 encode critical functions (Byram *et al*., 2004). To examine which additional cp26 genes are important for *in vitro* growth, 17 previously uncharacterized cp26 ORFs were targeted for inactivation by allelic exchange (Fig. 1 and Tables 1 and 2). These included all remaining ORFs on cp26 that had not previously been inactivated, except for the four genes encompassing the plasmid replication region (*bbb10–13*) and two small ORFs (*bbb20–21*) of 110 and 95 base pairs respectively. The limited coding sequences of *bbb20* and *bbb21*, and their lack of expression and conservation among other *B. burgdorferi* isolates (R. Byram, unpubl. data, Ojaimi *et al*., 2003; Glöckner *et al*., 2004), suggest that they are not protein-coding sequences. Gene inactivations were performed by independent transformation of *B. burgdorferi* strain B31 clone A (B31-A) with 12 different suicide plasmids designed for targeted allelic exchange with single genes or several adjacent genes (Table 1 and Fig. 1B). The allelic exchange event for each mutant was verified by polymerase chain reaction (PCR) with primers specific to the individual gene, followed by confirmation via Southern blot analysis (data not shown). Although the frequencies with which mutants were obtained varied for different constructs, all 17 remaining cp26 ORFs could be inactivated in some context (Table 2). Inactivation of each of these genes indicated that, unlike *resT*, they are not individually required for *B. burgdorferi* growth *in vitro*. This outcome was unanticipated because of our previous inability to displace cp26 with an incompatible plasmid carrying only *resT* (Byram *et al*., 2004). These conflicting results could be explained, however, if an essential function carried by cp26 were redundantly fulfilled by more than one cp26 gene, allowing deletion of each gene singly, whereas simultaneous deletion of all these genes would be lethal for the spirochete. Alternatively, distinct genes could encode important but unrelated functions, and the additive negative effect of their combined loss might render the spirochete non-viable.

**Table 2:** Mutant alleles on cp26.

	Strains recovered		
		
Gene(s) targeted for inactivation^a^	Merodiploid	Mutant	Reference
BBB01–2	–	+	This study
BBB03 (*resT*)	+	–	Byram *et al*. (2004)
BBB04 (*chbC*)	–	+	Tilly *et al*. (2001)
BBB05–7	–	+	This study
BBB08–9	–	+	This study
BBB14–15	–	+	This study
BBB16 (*oppAIV*)	–	+	Bono *et al*. (1998)
BBB17 (*guaB*)	–	+	Tilly *et al*. (1998)
BBB18 (*guaA*)	–	+	This study
BBB19 (*ospC*)	–	+	Tilly *et al*. (1997)
BBB22–23	–	+	This study
BBB24–27	+	–	This study
BBB24–25	–	+	This study
BBB26–27	+	–	This study
BBB26	+	+^b^	This study
BBB27	±^c^	+	This study
BBB28	–	+	This study
BBB29	–	+	Byram *et al*. (2004)

a As described in Table 1, Fig. 1 and *Experimental procedures*.

b Merodiploid was resolved to a mutant following passage in liquid and solid media under antibiotic selection.

c Mutants were readily obtained in the wild-type genetic background, whereas the merodiploid with pRB3 was resolved to a mutant only following passage in liquid and solid media under antibiotic selection.

**Table 1:** Inactivation of cp26 ORFS in B31-A.

1 Gene designation and coordinates (1–26498)	2 Primers used to amplify gene(s)^a^	3 Deletion region	4 Number of base pairs deleted	5 Inactivation plasmid	6 Primers used for inactivation	7 Antibiotic resistance gene used	8 Primers used to screen transformants
BBB01 (16–321)	1 and 2	32–686	654	XL-BBB01–02Δ	3 and 4	*aacC1*	1 and 2
BBB02 (751–311)	1 and 2	32–686	654	XL-BBB01–02Δ	3 and 4	*aacC1*	1 and 2
*chbA* (4428–4084)	5 and 6	3951–4788	837	XL-BBB05–07Δ	7 and 8	*kan*	5 and 6
*chbB* (4754–4440)	5 and 6	3951–4788	837	XL-BBB05–07Δ	7 and 8	*kan*	5 and 6
BBB07 (4769–5863)	5 and 6	3951–4788	837	XL-BBB05–07Δ	7 and 8	*kan*	5 and 6
BBB08 (6517–5891)	9 and 10	6054–7096	1042	XL-BBB08–09Δ	11 and 12	*kan*	9 and 10
BBB09 (6677–7711)	9 and 10	6054–7096	1042	XL-BBB08–09Δ	11 and 12	*kan*	9 and 10
BBB14 (11417–10923)	13 and 14	11014–12011	997	XL-BBB14–15Δ	15 and 16	*aacC1*	13 and 14
BBB15 (11636–11737)	13 and 14	11014–12011	997	XL-BBB14–15Δ	15 and 16	*aacC1*	13 and 14
guaA (16718–15135)	41 and 42	15926–16159	233	XL-*guaA*Δ	17 and 18	*aacC1/kan*	41 and 42
BBB22 (19321–17969)	19 and 20	18565–19949	1384	XL-BBB22–23Δ	21 and 22	*aacC1*	19 and 20
BBB23 (20822–19434)	19 and 20	18565–19949	1384	XL-BBB22–23Δ	21 and 22	*aacC1*	19 and 20
BBB24–27 (21364–22606)	23 and 24	20897–23641	2744	XL-BBB24–27Δ	NA	*aacC1*	31 and 32
BBB24 (21364–20861)	23 and 24	20897–21639	742	XL-BBB24–25Δ	NA	*aacC1*	32 and 33
BBB25 (21851–21342)	23 and 24	20897–21639	742	XL-BBB24–25Δ	NA	*aacC1*	32 and 33
BBB26 (21898–22590)	26 and 32	21923–22579	656	XL-BBB26Δ	37 and 39	*aacC1*	35 and 36
BBB27 (23154–22606)	26 and 32	22653–23091	438	XL-BBB27Δ	36 and 40	*aacC1*	33 and 34
BBB26–27 (21898–23154)	52 and 53	21923–23091	1168	XL-BBB26–27Δ	39 and 40	*aadA*	33 and 34
BBB28 (23255–24496)	24 and 25	23564–24037	473	XL-BBB28Δ	26 and 27	*aacC1/kan*	24 and 25

a DNA fragment used in subsequent cloning of allelic exchange construct for targeted gene inactivation.

NA, not applicable.

**Fig. 2:**
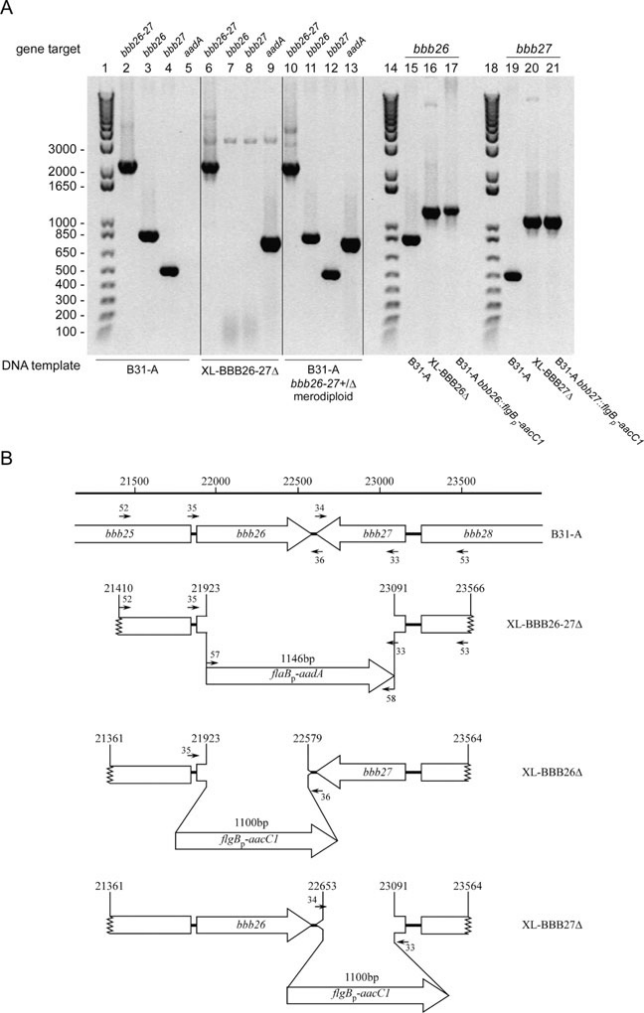
A. PCR analysis of genomic DNA from *B. burgdorferi* clones transformed with gene inactivation constructs targeting genes in the *bbb26–27* region. Template DNAs from transformants are identified below the lanes and PCR amplification targets above the lanes. Template DNA from B31-A illustrates the PCR products from the wild-type alleles of *bbb26–27*, *bbb26* and *bbb27* (lanes 2–5, 15 and 19), whereas template DNAs from the gene inactivation constructs (XL-BBB26–27Δ, XL-BBB26Δ and XL-BBB27Δ) depict the PCR profiles of the mutated alleles (lanes 6–9, 16 and 20). The PCR products resulting from the clones transformed with the allelic exchange inactivation plasmids are illustrated in lanes 10–13, 17 and 21. The 1 kbp-plus size standards (Invitrogen) were run in lanes 1, 14 and 18 and sizes (base pairs) are indicated to the left of the panel. B. Graphical representation of the *bbb26–27* region on cp26 (B31-A) and the cloned pieces of DNA used for the allelic exchange constructs (XL-BBB26–27Δ, XL-BBB26Δ and XL-BBB27Δ). The 1168 bp region of cp26 between nucleotides 21923 and 23091 was replaced with the 1146 bp *flaB*_p_–*aadA* resistance cassette to create XL-BBB26–27Δ for disruption of both *bbb26* and *bbb27*. The 742 bp region of cp26 between nucleotides 21923 and 22579 was replaced with the 1100 bp *flgB*_p_–*aacC1* resistance cassette to create XL-BBB26Δ for disruption of *bbb26*. The 438 bp region of cp26 between nucleotides 22653 and 23091 was replaced with the 1100 bp *flgB*_p_–*aacC1* resistance cassette to create XL-BBB27Δ for disruption of *bbb27*. Locations of the primers used for analysis in (A) are indicated and the sequences are listed in Table S1.

Several pieces of data were consistent with the presence of critical but complementary genes on cp26. Mutations in such genes should be recoverable as single, but not double, mutants. Many genes on cp26 could be inactivated in conjunction with adjacent genes (Table 2). However, repeated attempts to delete the cp26 region encompassing *bbb24–27* failed, yielding only merodiploid transformants in which both wild type and mutant alleles of these four genes were present in the same bacterium (as depicted in Fig. 1C), similar to the outcome of previous transformations in which the essential *resT* gene was targeted for inactivation (Table 2) (Byram *et al*., 2004). Subsequent attempts to inactivate just *bbb24–25* from this region easily produced mutants lacking these genes (Table 2). We also obtained single mutants in both *bbb26* and *bbb27* when these genes were targeted individually (Table 2). In contrast, only merodiploid transformants were recovered following electroporation with a construct designed to delete both *bbb26* and *bbb27* (Table 2). PCR analyses of the targeted loci of different mutants in the *bbb26–27* region illustrate the various outcomes of these transformations (Fig. 2). Additional analysis of the *bbb26–27* merodiploid transformants using primers external to the allelic exchange construct (primers 24 and 30, Table S1) along with primers within the *flaB*_p_–*aadA* resistance cassette (primers 55 and 56, Table S1) demonstrated that recombination had occurred at the *bbb26–*27 locus on cp26 (data not shown). These data suggest that together *bbb26* and *bbb27* encode an essential function(s) and that at least one or the other of these cp26 genes, in addition to *resT*, is required for bacterial growth.

### Displacement of cp26 by an incompatible plasmid harbouring *resT*, *bbb26* and *bbb27*

**Fig. 3:**
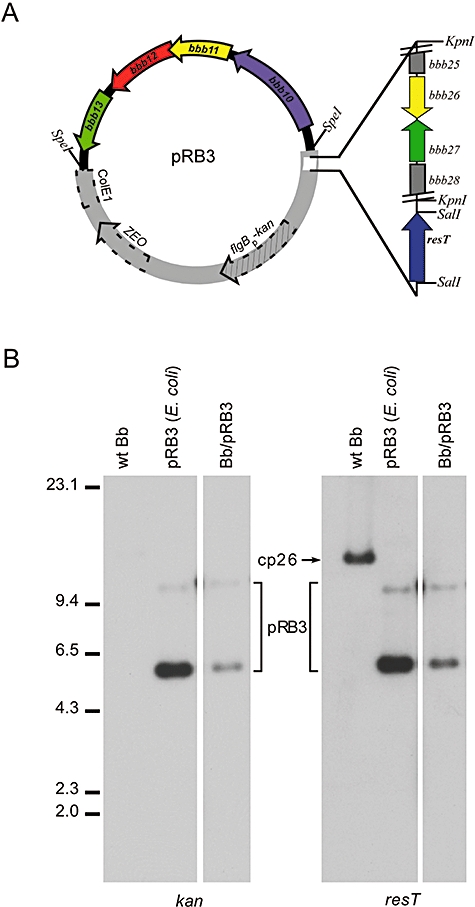
A. Graphical representation of pRB3, a cp26-incompatible shuttle vector that carries the *resT*, *bbb26* and *bbb27* genes of cp26. The *bbb25* and *bbb28* genes on pRB3 are truncated, lacking 3′ ends. Relevant restriction sites are shown. *colE1*, *E. coli* plasmid origin of replication; ZEO, zeocin-resistance marker; *flgB*_P_–*kan*, kanamycin resistance cassette; *bbb10–13*, ORFs from cp26 that confer autonomous plasmid replication and incompatibility with cp26 (Byram *et al*., 2004). B. Displacement of cp26 from *B. burgdorferi* by pRB3 as demonstrated by Southern blot analysis of transformants. Wild-type *B. burgdorferi* DNA (wt Bb), *E. coli* plasmid DNA of shuttle vector carrying *resT*, *bbb26* and *bbb27* (pRB3) and total genomic DNA from *B. burgdorferi* transformed with pRB3 (Bb/pRB3). Southern blot probed with the kanamycin resistance gene (left panel), which is present on pRB3, or the same blot stripped and reprobed with the *resT* gene (right panel), which is present on both cp26 and pRB3. An arrow indicates the supercoiled form of endogenous cp26; brackets denote supercoiled and nicked forms of pRB3. The mobilities of size standards (kbp) are indicated to the left of the panels.

Previous experiments with shuttle vectors derived from non-essential plasmids of *B. burgdorferi* demonstrated specific displacement of the endogenous plasmid from which the replication/incompatibility region of a vector originated (Stewart *et al*., 2001; 2003; Eggers *et al*., 2002; Grimm *et al*., 2004b). We assumed therefore that we would likewise be able to displace cp26 with an incompatible vector, provided that it harboured all cp26 genes that encode essential functions. The results described earlier suggested that either the *bbb26* or the *bbb27* gene product is critical for *B. burgdorferi* viability, in addition to *resT*. To examine this hypothesis, we constructed the vector pRB3, a modified version of the cp26-incompatible plasmid pBSV26 (Byram *et al*., 2004), into which we cloned *resT*, *bbb26* and *bbb27* (Fig. 3A).

We next attempted to displace cp26 by transforming B31-A with pRB3. Southern blot analysis of undigested genomic DNA demonstrated that cp26 was absent from pRB3 transformants (Fig. 3B). We conclude that the inclusion on pRB3 of essential functions encoded by *resT*, *bbb26* and *bbb27*, in conjunction with the plasmid maintenance and incompatibility functions conferred by the *bbb10–13* locus, permitted displacement of the endogenous cp26 plasmid of *B. burgdorferi* for the first time.

As described earlier, we found that either *bbb26* or *bbb27* could be inactivated in a strain carrying cp26, but that *bbb26–27* double mutants were never recovered in this genetic background. We wondered if this restriction still applied to *B. burgdorferi* lacking cp26, or if both *bbb26* and *bbb27* were required when other cp26 genes were absent. To address this question, we attempted to individually and doubly inactivate both *bbb26* and *bbb27* in a strain in which cp26 had been displaced by pRB3. Single *bbb26* or *bbb27* mutants were each recovered in this background (data not shown and Table 3). However, no double *bbb26–27* mutants were ever obtained in the strain lacking cp26, but harbouring pRB3. Likewise, attempts to displace pRB3 with pBSV26*resT*G, an incompatible vector carrying only *resT* (and a different selectable marker) were unsuccessful. These results further confirmed that *bbb26* and *bbb27* provide critical but complementary functions, and together with *resT*, determine the essential nature of cp26 in *B. burgdorferi*.

**Table 3:** Attempted displacement of cp26 by incompatible plasmids carrying distinct cp26 gene complements.

Strain	cp26	cp26-incompatible shuttle vector	Reference
B31-A	+	None	Bono *et al*. (2000)
BbRB1^a^	coexisting	pBSV26	Byram *et al*. (2004)
BbRB2^b^	coexisting	pBSV26*resT*	Byram *et al*. (2004)
BbRB3	Displaced	pRB3 (pBSV26*resTbbb26–27*)	This study
BbRB4	Displaced	pRB3Δ*bbb26*^c^	This study
BbRB5	Displaced	pRB3Δ*bbb27*^d^	This study

a Strain previously denoted B31-A/pBSV26.

b Strain previously denoted B31-A/pBSV26resT.

c *bbb26* on pRB3 inactivated by allelic exchange with a construct carrying flgB_p_–aacC1.

d *bbb27* on pRB3 inactivated by allelic exchange with a construct carrying flgB_p_–aacC1.

### Altered growth of cp26 mutants

**Fig. 4:**
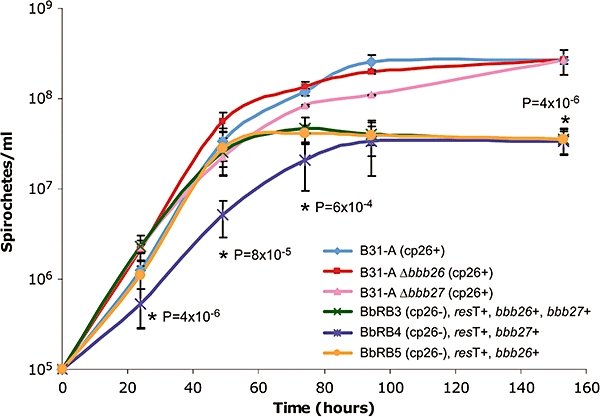
*In vitro* growth of *B. burgdorferi* strains with different cp26 gene complements. The presence and absence of cp26, *resT*, *bbb26* and *bbb27* from B31-A-derivative strains are as indicated. The densities of cultures from a starting dilution of 1 × 10^5^ spirochetes ml^−1^ were determined every 24 h using a Petroff–Hausser counting chamber. Data are presented as averages of at least three separate experiments, with triplicate cultures per strain in each experiment, and error bars represent standard deviation from the mean. Statistical analyses (Student's *t*-test, two tailed) were used to compare pairs of data points. The exponential phase growth of BbRB4 was significantly slower than that of all other strains, as indicated by asterisks at 24, 49 and 72 h; the *P*-values represent comparisons with BbRB3. All three strains lacking cp26 reached significantly lower stationary phase densities relative to B31-A, as indicated by the asterisk and *P*-value at 153 h.

Although our data indicate that only the *resT* and *bbb26* or *bbb27* genes of cp26 are absolutely required for bacterial viability, mutants in which cp26 was displaced and *bbb26* or *bbb27* was inactivated were not easily recovered. These transformations initially yielded merodiploids, which only resolved into the ‘clean’ allelic exchange mutants described earlier after subsequent passage and replating under antibiotic selection. This suggested that mutants in which cp26 had been displaced and that carried only one of the *bbb26–27* gene pair were at a selective growth disadvantage relative to wild-type organisms. To address this possibility, we compared the growth in liquid and solid media of several strains (Fig. 4).

Consistent with the ability to recover single *bbb26* and *bbb27* mutants in the wild-type B31-A genetic background, no difference in growth rate or maximum stationary phase density in liquid medium was observed between B31-A and spirochetes lacking either *bbb26* (B31-A, Δ*bbb26*, cp26^+^) or *bbb27* (B31-A, Δ*bbb27*, cp26^+^) (Fig. 4). The BbRB3 strain (B31-A, cp26^–^, *resT*^+^, *bbb26*^+^, *bbb27*^+^), which lacks cp26 but contains *resT*, *bbb26* and *bbb27* (Table 3), displayed a doubling time similar to B31-A (6.1 h), but only reached an average stationary phase density of ∼4 × 10^7^ spirochetes ml^−1^ (compared with ∼3 × 10^8^ spirochetes ml^−1^ for B31-A), even after 120 h of growth (Fig. 4). BbRB3 and BbRB5 (B31-A, cp26^–^, *resT*^+^, *bbb26*^+^), the isogenic derivative lacking *bbb27*, demonstrated similar growth rates (Fig. 4). However, the isogenic strain lacking *bbb26* (BbRB4) (B31-A, cp26^–^, *resT*^+^, *bbb27*^+^) grew at a slower rate than the other two mutant strains, with a doubling time of 9.5 h, and reached a comparable stationary phase density (3.5 × 10^7^ spirochetes ml^−1^) approximately 20 h later (Fig. 4). Conversely, BbRB5 colony formation in solid medium was delayed up to 7 days relative to BbRB3 and BbRB4. The growth defects of BbRB4 and BbRB5 in liquid and solid media, respectively, are consistent with the difficulty with which these mutants were initially recovered.

### Predicted cellular locations of the BBB26 and BBB27 proteins

Given the requirement for *bbb26* and *bbb27* by *B. burgdorferi*, we were interested in the predicted proteins that these genes encode. BBB26 and BBB27 are convergently transcribed ORFs (Fig. 1A) that encode unrelated basic proteins of 27.2 kDa (pI 9.2) and 21.5 kDa (pI 9.5), respectively. Both genes are described in the *B. burgdorferi* genome annotation (Fraser *et al*., 1997; Casjens *et al*., 2000) as hypothetical ORFs that lack sequence homology with genes in other organisms. The BBB26 and BBB27 ORFs are conserved, however, in the closely related Lyme disease spirochete *Borrelia garinii*, in which the predicted proteins share ∼90% amino acid identity with the *B. burgdorferi* homologues (Glöckner *et al*., 2004). No differences in protein profiles were detected when cellular lysates of *bbb26* and *bbb27* mutants were compared with wild-type B31-A on a silver-stained, single dimension SDS-polyacrylamide gel (data not shown), suggesting that, although critical, these proteins are not abundant components of *in vitro* grown organisms, a trait shared with ResT (R. Byram, unpubl. data).

Computational analyses of the BBB26 and BBB27 open reading frames suggested that both proteins could be membrane-associated. While BBB26 lacks a predicted amino-terminal signal sequence, Psort analysis (Nakai and Horton, 1999) identified hydrophobic residues 24–42 of BBB26 as a probable single transmembrane region, potentially anchoring the largely hydrophilic protein to the cytoplasmic membrane and localizing a majority of the protein to the periplasmic space. Psort analysis of BBB27 identified residues 1–22 as an amino-terminal signal sequence, but predicted that this leader peptide would be uncleaved. However, recent data suggest that Psort has failed to identify nearly half of the experimentally confirmed spirochetal lipoprotein sequences (Setubal *et al*., 2006). Because of the inaccuracy of Psort with regards to spirochete sequences, a novel spirochete-specific lipoprotein prediction algorithm, SpLip, has been developed from a confirmed spirochetal lipoprotein data set (Setubal *et al*., 2006). SpLip analysis identified BBB27 as a canonical lipoprotein with a characteristic spirochetal lipobox (LFYGC) (Setubal *et al*., 2006). Consistent with the findings of another lipoprotein signal peptide prediction algorithm LipoP (Juncker *et al*., 2003), SpLip predicted cleavage of the signal peptide between residues 15 and 16 (LLFYG/CSTIS), followed by amino-terminal acylation of BBB27 at the N-terminal Cys residue and association with either the spirochete inner or the outer membrane (Setubal *et al*., 2006).

**Fig. 5:**
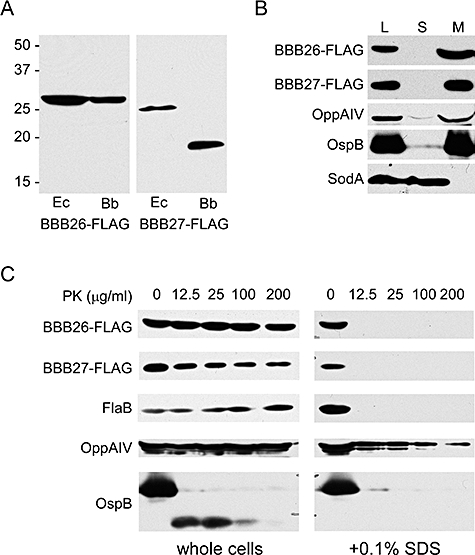
Expression and cellular localization of the BBB26-FLAG and BBB27-FLAG proteins. A. Proteins lysates from *E. coli* (Ec) or *B. burgdorferi* B31-A34 (Bb) harbouring either pBSV2ex *bbb26*-FLAG or pBSV2ex *bbb27*-FLAG were separated by SDS-PAGE and analysed by immunoblot with anti-FLAG antibodies. The mobilities of size standards (molecular weights in kDa) are indicated to the left of the figure. B. Protein lysates from *B. burgdorferi* clone A34 harbouring either pBSV2ex *bbb26*-FLAG or pBSV2ex *bbb27*-FLAG were harvested and separated into soluble and membrane fractions by ultracentrifugation. Protein fractions from equivalent numbers of spirochetes were subjected to SDS-PAGE and analysed by immunoblot with FLAG (BBB26 and BBB27), OppAIV (inner membrane), OspB (outer membrane) and SodA (cytoplasmic) antisera. Representative results for the localization of the OppAIV, OspB and SodA proteins from *B. burgdorferi* clone A34 harbouring either pBSV2ex *bbb26*-FLAG or pBSV2ex *bbb27*-FLAG are shown. L, total cell lysate; S, soluble protein fraction; M, membrane protein fraction. C. Equal numbers of whole or 0.1% SDS-treated (+0.1% SDS) cells of *B. burgdorferi* clone A34 harbouring either pBSV2ex *bbb26*-FLAG or pBSV2ex *bbb27*-FLAG were incubated with different concentrations (μg ml^−1^) of proteinase K (PK). Lysates of PK-treated bacteria were separated by SDS-PAGE and analysed by immunoblot with FLAG (BBB26 and BBB27), flagellin (FlaB, periplasmic marker), OppAIV (inner membrane marker) and OspB (surface exposed, outer membrane marker) antisera. Representative results for the proteinase K sensitivity of the FlaB, OppAIV and OspB proteins from *B. burgdorferi* clone A34 harbouring either pBSV2ex *bbb26*-FLAG or pBSV2ex *bbb27*-FLAG are shown.

In an initial attempt to experimentally address the computational predictions that both the BBB26 and BBB27 proteins are membrane-associated, polyclonal rabbit antisera were elicited against the recombinant proteins purified from insoluble inclusion bodies in *Escherichia coli.* However, the antibodies failed to recognize any proteins in *B. burgdorferi* lysates (data not shown). This suggested that either the epitopes of the denatured proteins were not exposed on the native proteins or that BBB26 and BBB27 were present in too low abundance in *B. burgdorferi* lysates to be detected by immunoblot analysis. To circumvent both of these potential limitations, *bbb26* and *bbb27* were cloned into the *Borrelia* expression vector pBSV2ex (Guyard *et al*., 2006) under the control of a strong *Borrelia* promoter (*flgB*_p_) and tagged with a C-terminal FLAG epitope (DYKDDDDK) (Hopp *et al*., 1988) to be used for immunoblot analysis. As expected, a protein of approximately 28 kDa was detected with monoclonal antiserum recognizing the FLAG epitope in total cell lysates of both *E. coli* and *B. burgdorferi* harbouring pBSV2ex *bbb26*-FLAG (Fig. 5A) and a protein of approximately 23 kDa was detected in total cell lysates of *E. coli* carrying pBSV2ex *bbb27*-FLAG (Fig. 5A). Consistent with the presence of an N-terminal signal peptidase II cleavage site in the BBB27 protein, a smaller protein of approximately 18 kDa was detected in total cell lysates of *B. burgdorferi* carrying the same construct (Fig. 5A), suggesting that BBB27 was processed when synthesized in *B. burgdorferi* but not when it was synthesized in *E. coli*. This may indicate a potential difference in the active site substrate specificity of the type II signal peptidase of spirochetes relative to other major bacterial groups, as suggested by Setubal *et al*. (2006) and further reflected in the inaccuracy of the Psort algorithm in predicting spirochetal lipoproteins (Setubal *et al*., 2006). The size difference between BBB27 synthesized in *E. coli* versus *B. burgdorferi* appears greater than predicted by cleavage of the signal peptide alone (∼21 kDa); this may reflect additional processing of BBB27 in *B. burgdorferi*. No proteins were detected in total cell lysates of *B. burgdorferi* carrying the pBSV2ex plasmid alone (data not shown), indicating that the FLAG antibody specifically recognized the engineered FLAG epitopes on the BBB26-FLAG and BBB27-FLAG proteins. Together these data demonstrated that the BBB26-FLAG and BBB27-FLAG proteins are synthesized in *B. burgdorferi* and can be detected using antiserum that recognizes the FLAG epitope.

To investigate the predicted membrane association of BBB26 and BBB27, *B. burgdorferi* harbouring either pBSV2ex *bbb26*-FLAG or pBSV2ex *bbb27*-FLAG were disrupted by French press and separated into total lysate, soluble and membrane fractions (see *Experimental procedures*). Cellular fractions were resolved by SDS-polyacrylamide gel electrophoresis (PAGE) and immunoblotted with FLAG, OppAIV, OspB and SodA antisera. Both the BBB26-FLAG and BBB27-FLAG proteins were detected in the total lysate and membrane fractions, but not the soluble fraction (Fig. 5B), as were the membrane proteins OppAIV and OspB. As anticipated, the cytoplasmic protein SodA (Fraser *et al*., 1997; Whitehouse *et al*., 1997) was limited to the soluble fraction (Fig. 5B). Together these data, along with the computational analysis, indicate that BBB26 and BBB27 are membrane-associated proteins.

To determine if either protein was surface exposed, intact borreliae harbouring either pBSV2ex *bbb26*-FLAG or pBSV2ex *bbb27*-FLAG were treated with varying amounts of proteinase K and bacterial lysates were immunoblotted with FLAG antiserum (Fig. 5C). Flagellin (FlaB), the major constituent of the flagella, was used as a periplasmic marker (Barbour *et al*., 1986; Bono *et al*., 1998; Bunikis and Barbour, 1999). The oligopeptide permease lipoprotein, OppAIV, served as a tightly associated inner membrane marker (Bono *et al*., 1998; Schulze and Zuckert, 2006), and the outer membrane lipoprotein OspB was used as a marker for surface exposed proteins (Bergström *et al*., 1989; Bono *et al*., 1998; Bunikis and Barbour, 1999). Little to no degradation of the BBB26-FLAG or the BBB27-FLAG protein was detected for intact cells across all concentrations of proteinase K, similar to the pattern observed for the internal proteins FlaB and OppAIV (Fig. 5C). In contrast, the surface exposed lipoprotein OspB was sensitive to degradation at the lowest concentration of proteinase K (12.5 µg ml^−1^, Fig. 5C), relative to untreated intact cells. Lysis of the borreliae with 0.1% SDS resulted in complete susceptibility of the BBB26-FLAG, BBB27-FLAG and FlaB proteins to protealysis (Fig. 5C), demonstrating that neither BBB26 nor BBB27 is intrinsically protease resistant. Conversely, only a slight increase in protease susceptibility was detected for OppAIV in SDS-treated lysates, relative to intact cells (Fig. 5C), suggesting that the proteinase K cleavage sites of this protein may have remained inaccessible despite the presence of detergent and/or this protein is resistant to proteolysis by proteinase K. OspB maintained sensitivity to proteolysis in SDS-treated lysates (Fig. 5C). Together these data suggest that neither BBB26-FLAG nor BBB27-FLAG is exposed on the *B. burgdorferi* outer surface and both are likely to be membrane-associated proteins localized to the periplasmic space.

Although a blast search of the current database did not identify any sequence homologues of BBB26 or BBB27, COG analysis revealed that both proteins share some amino acid similarity to peptide-cleaving enzymes (Altschul *et al*., 1990; Marchler-Bauer *et al*., 2005). However, no protease activity was detected when lysates of *B. burgdorferi* harbouring either pBSV2ex *bbb26*-FLAG or pBSV2ex *bbb27*-FLAG were analysed on casein zymogram gels or in spectrophotometric protease assays which used casein as a substrate, in contrast to lysates of *B. burgdorferi* producing a previously identified protease of *Borrelia* (Guyard *et al*., 2006) (data not shown).

## Discussion

In this study we defined the full complement of genes carried by cp26 that are essential for spirochete survival *in vitro.* These include *resT*, a telomere resolvase (Kobryn and Chaconas, 2002; Byram *et al*., 2004), and either *bbb26* or *bbb27*, adjacent genes unique to *Borrelia*. This study used two different genetic techniques to identify those genes encoded on cp26 that are crucial for *B. burgdorferi* growth: targeted gene inactivation by allelic exchange and plasmid displacement. Allelic exchange allows targeted deletion of a specific gene by replacement with a selectable marker, via homologous recombination with sequences flanking the targeted gene (Fig. 1B and C). Using this method, non-essential genes can be inactivated. Conversely, inactivation of essential genes would result in a lethal phenotype and mutants would not be recovered. An alternative outcome is merodiploid formation, in which both wild-type and mutated forms of the gene coexist in the same organism (Fig. 1C), suggesting that loss of the targeted gene is deleterious for the spirochete. Merodiploids may be resolved to allelic exchange mutants following repeated passage in the presence of selection if the target gene is not absolutely required. Therefore, failure to recover mutants using this method suggests the critical presence of the targeted gene for the *in vitro* survival of *B. burgdorferi*.

Plasmid displacement results in simultaneous loss of a number of genes by removal of an entire plasmid. This method asks whether the gene(s) present on an incompatible plasmid allow(s) displacement of the endogenous complement of genes carried on a particular replicon, suggesting that the reduced set of genes is sufficient for survival. Two plasmid species are considered incompatible when they contain identical replication and/or partitioning functions, resulting in the loss of one of the plasmids (Austin and Nordström, 1990). However, loss of one of the plasmids is not the only observed outcome of competition between incompatible plasmids. We previously demonstrated that the plasmid pBSV26 harbouring the cp26 replication machinery, as well as pBSV26*resT*, the same plasmid carrying the *resT* gene, when introduced into *B. burgdorferi* either coexisted with the endogenous cp26 or integrated into a cp26 replicon (Byram *et al*., 2004). Therefore, unless the minimal set of genes present on the introduced plasmid is sufficient for *in vitro* survival of *B. burgdorferi*, displacement will not be observed. In the current study, both targeted gene inactivation by allelic exchange and plasmid displacement suggest the central roles of *bbb26* and *bbb27*, in addition to *resT* (Byram *et al*., 2004), for *B. burgdorferi* growth *in vitro*.

Strains lacking cp26, but harbouring only *resT*, *bbb26* and *bbb27*, were impaired in growth compared with the parent B31-A strain (Fig. 4). These clones are lacking 22 of the 29 cp26 genes, including *guaA*, *guaB*, *oppAIV*, *bbb22*, *bbb23* and *bbb29*, which likely play significant roles in the salvage of purines, peptides and sugars, functions that could be important for spirochete growth *in vitro* and/or *in vivo* (Margolis *et al*., 1994; Zhou *et al*., 1997; Bono *et al*., 1998; Botkin *et al*., 2006). Inactivation of *bbb26* in the strain in which cp26 had been displaced by pRB3, carrying *resT*, *bbb26* and *bbb27*, resulted in an even greater growth impairment in liquid medium compared with the parent (Fig. 4). In addition, deletion of *bbb27* from pRB3 led to a delay in colony formation relative to the parent clone, reaffirming the physiological importance of the *bbb26* and *bbb27* gene products for *B. burgdorferi* growth.

The cp26 genes *bbb26* and *bbb27* appear to be required for *B. burgdorferi* survival *in vitro*, yet their functions remain unknown. Both genes are unique to *Borrelia* and neither shares convincing sequence similarity with genes of known function. Despite the lack of homology to genes from other bacteria, the BBB26 protein does contain a conserved domain of unknown function (DUF955) found among a number of bacterial and viral proteins. The BBB26 protein also demonstrates limited sequence similarity to members of COG 2856, a family of predicted zinc peptidases (Marchler-Bauer *et al*., 2005). Similar to BBB26, BBB27 displays limited sequence similarity to peptide-cleaving enzymes. Most notably, it shares some sequence similarity to protease-1 from the fungal pathogen *Pneumocystis* and to a probable peptidase from *Rickettsia prowazekii* (Altschul *et al*., 1990). If BBB26 or BBB27 does have peptidase activities, neither can use casein as a substrate, as no band of clearing was detected on a casein zymogram and no protease activity was detected by spectrophotometric assay (data not shown), suggesting that the putative peptidase activity(s), if any, of these proteins are not as general proteases and may be specific to peptides of a particular sequence or size.

Computational predictions as well as direct analysis of fractionated *B. burgdorferi* cells suggested that BBB26 and BBB27 are associated with a spirochetal membrane (Fig. 5B). Protease accessibility assays demonstrated that neither protein was exposed on the spirochete outer surface (Fig. 5C). BBB27 has a consensus spirochetal lipoprotein signal sequence (Setubal *et al*., 2006) that appears to be processed in *B. burgdorferi* (Fig. 5A) and therefore, similar to OppAIV (Bono *et al*., 1998; Schulze and Zuckert, 2006), may be a periplasmic protein tethered to the cytoplasmic membrane by an N-terminal acyl group. BBB26 is predicted to harbour a single transmembrane region that could allow association with the cytoplasmic membrane and potential periplasmic localization. Together these data suggest that both proteins may be present in the periplasmic space. Given its small size, C-terminal location and successful use in other systems (Molloy *et al*., 1994; Yamamoto *et al*., 2003; Mura *et al*., 2004), it is unlikely that the FLAG epitope tag used for these experiments interfered with the expression or misdirected the cellular localization of BBB26 and BBB27.

The uniquely segmented *B. burgdorferi* genome can contain as many as 23 genetic elements at one time (Stevenson *et al*., 1996; 1998; Fraser *et al*., 1997; Casjens *et al*., 2000; Miller *et al*., 2000). The reason that the genome of this bacterium is divided among such a large variety of replicons remains unclear. However, it appears that the distribution of genes across the numerous *B. burgdorferi* replicons, and on cp26 in particular, has not occurred at random. While cp26 carries genes important for bacterial growth, it also harbours a gene required for mammalian infectivity. Presumably, the linkage of the essential virulence gene *ospC*, which is only required at one specific stage of the *B. burgdorferi* life cycle (Grimm *et al*., 2004a; Stewart *et al*., 2006; Tilly *et al*., 2006; 2007), with constitutively required functions encoded by *resT*, *bbb26* and *bbb27*, assures that this crucial virulence factor will not be lost during growth in environments in which *ospC* does not provide a selective advantage. Indeed, we found that when the *ospC* gene is carried by a non-essential plasmid, it was lost by spirochetes in infected mice subsequent to the development of the host acquired immune response (Tilly *et al*., 2006; 2007). Consistent with this observation, Xu and colleagues have also shown that persistent expression of *ospC* by *B. burgdorferi* in an infected mammal is deleterious to the spirochete (Xu *et al*., 2007). Although *B. burgdorferi* lacking *ospC* are efficiently acquired by feeding ticks, loss of OspC precludes infection of a new mammalian host during the next tick blood meal, thereby aborting the infectious cycle (Tilly *et al*., 2006; 2007). This demonstrates that *ospC* must be carried by an essential genetic element, such as cp26 or the chromosome, in order to assure its retention by the spirochete in the mammalian host. In sum, it appears that genetic linkage of critical physiological and virulence functions on cp26 has resulted in its stable maintenance throughout the evolution of *B. burgdorferi*.

In a previous study we showed that cp26 encodes functions essential to *B. burgdorferi* viability including, but not limited to, the telomere resolvase *resT* (Byram *et al*., 2004). Herein we conclude that in addition to *resT*, the membrane-associated proteins encoded by the cp26 genes *bbb26* and *bbb27* are important for spirochete viability, as the cp26 plasmid could only be displaced by an incompatible plasmid when it carried these three genes, and a mutant lacking both *bbb26* and *bbb27* was never obtained. Future studies are required to identify the contribution of *bbb26* and *bbb27* to basic spirochete physiology, both *in vitro* and *in vivo*. Together, the *resT*, *bbb26* and *bbb27* genes contribute to spirochete survival and thereby help to ensure the faithful maintenance of the cp26 replicon throughout the varying conditions encountered in the mouse-tick infectious cycle.

## Experimental procedures

### *B. burgdorferi* strains and growth conditions

*B. burgdorferi* strains were cultivated at 35°C either in liquid BSK-H complete medium (Sigma) or in Barbour-Stoenner-Kelly (BSKII) medium containing gelatin and supplemented with 6% rabbit serum (Pel-Freez Laboratories). *B. burgdorferi* cultures were plated in solid BSKII medium and grown at 35°C with 2.5% CO_2_ (Rosa *et al*., 1996). Kanamycin was used at a concentration of 200 μg ml^−1^, gentamicin at 40 μg ml^−1^ and streptomycin at 50 μg ml^−1^. B31 clone A (B31-A) (Bono *et al*., 2000) is a non-infectious derivative of type strain B31 (ATCC 35210), which was isolated from a tick collected on Shelter Island in New York (Burgdorfer *et al*., 1982). B31 clone A34 (A34) is a derivative of B31-A that also lacks lp56. For growth rate comparisons, strains were inoculated from freezer stocks into 5 ml of BSKII containing the appropriate antibiotic and grown to an approximate density of 1 × 10^7^ spirochetes ml^−1^. Strains were subsequently diluted in triplicate to 1 × 10^5^ spirochetes ml^−1^ in 5 ml of BSKII containing the appropriate antibiotic(s). Spirochete density was determined every 24 h using a Petroff–Hausser counting chamber. Time for colony formation was assessed by diluting various strains to 1 × 10^3^ spirochetes ml^−1^ and plating 100 μl of culture in solid BSKII medium on duplicate plates. The plates were observed daily for about 18 days for appearance of colonies. Colony formation analysis was blinded and performed twice.

### Constructs for gene inactivation by allelic exchange

The primer sequences used in this study were based on the previously described B31 genome sequence (Fraser *et al*., 1997; Casjens *et al*., 2000) and are shown in Table S1. The majority of the allelic exchange constructs for inactivation of cp26 genes were cloned by the following strategy, as diagramed in Fig. 1B. Details for each inactivation construct are provided in Table 1. A region of cp26 spanning the targeted gene(s) was amplified from B31-A genomic DNA (primers listed in column 2 of Table 1 and Table S1), using either *Taq* polymerase (fragments < 2.5 kb) (New England Biolabs) or the Expand Long Template PCR system (fragments > 2.5 kb) (Roche Molecular Biochemicals), and cloned into the vector pCR-XL-TOPO (Invitrogen). A deletion in each targeted gene(s) (as described in column 3 of Table 1) was constructed by inverse PCR performed with the primers listed in column 6 of Table 1, all of which introduce SalI sites, and the Expand Long Template PCR system. The *flgB*_P_–*aacC1* gene cassette (Elias *et al*., 2003), which confers gentamicin resistance, was amplified using *Taq* polymerase and primers 43 and 44 and cloned into the pCR2.1-TOPO vector (Invitrogen). The *flgB*_P_–*aacC1* gene cassette was removed from the pCR2.1-TOPO vector by XhoI digestion and ligated into inactivation constructs digested with SalI to create individual gene deletion plasmids. The *flgB*_P_–*kan* gene cassette (Bono *et al*., 2000), which confers kanamycin resistance, was amplified with *Taq* polymerase and primers 43 and 45 and cloned into the pCR2.1-TOPO vector. Inactivation constructs to confer kanamycin-resistance were created as above, except with a *flgB*_P_–*kan* fusion. The *flaB*_P_–*aadA* fusion, which confers spectinomycin/streptomycin resistance, was generated by PCR-amplifying the *flaB* promoter region from B31-A, using primers 50 and 54, and the *aadA* gene from pKFSS1 (Frank *et al*., 2003), using primers 55 and 56. Both the *flaB* and *aadA* DNA fragments were digested with NdeI, purified, ligated together and cloned into the pCR2.1-TOPO vector (Invitrogen). The *flaB*_P_–*aadA* cassette was subsequently PCR-amplified using primers 57 and 58, digested with XhoI and ligated into the appropriate SalI-digested inactivation construct. The structures of all plasmids were confirmed by DNA sequencing using the ABI Big Dye Terminator Cycle Sequencing Ready Reaction Kit (PE Applied Biosystems) on an ABI 3700 DNA sequencer.

Two constructs used for gene inactivation by allelic exchange were created by slightly different cloning strategies. To inactivate *bbb24–27*, a 4.9 kb region of cp26 including the full-length *bbb24*, *bbb25*, *bbb26* and *bbb27* genes was amplified from B31-A genomic DNA using the Expand Long Template PCR system and primers 23 and 24, and cloned into the pCR-XL-TOPO vector to create XL-*bbb24–27*. A 2.7 kb deletion in the *bbb24–27* genes encompassing cp26 nucleotides 20897–23641 was constructed by XmnI restriction enzyme digestion. The *flgB*_P_–*aacC1* gene cassette was amplified using *Taq* polymerase and primers 46 and 47, and cloned into the pCR2.1-TOPO vector. The *flgB*_P_–*aacC1* gene cassette was removed from the pCR2.1-TOPO vector by PvuII digestion and ligated into XL-*bbb24–27*Δ20897-23641 that had been digested with XmnI. To inactivate just *bbb24–25*, a 742 bp deletion encompassing nucleotides 20897–21639 in the *bbb*24 and *bbb*25 genes carried by the plasmid XL-*bbb24–27* was created by partial digestion of the plasmid with XmnI and SwaI restriction enzymes and then ligated with the *flgB*_P_–*aacC1* gene cassette removed from the pCR2.1-TOPO vector by PvuII digestion.

### Construction of shuttle vectors

To create pRB3 (pBSV26*resTbbb26–27*), a 3.4 kb region of cp26 encompassing the *bbb26* and *bbb27* genes was amplified using the Expand Long Template PCR system and primers 30 and 31, and cloned into the pCR-XL-TOPO vector. The 2.1 kb gene fragment including the *bbb26* and *bbb27* genes was subsequently removed from the pCR-XL-TOPO vector by KpnI digestion and ligated into the multiple cloning site of KpnI-digested pBSV26*resT* (Byram *et al*., 2004), to obtain pRB3.

To create pBSV26*resT*G, the *flaB*_P_–*aacC*1 resistance cassette was amplified using *Taq* DNA polymerase (New England Biolabs) and primers 50 and 51, and cloned into the pCR-XL-TOPO vector. The fragment was removed from the pCR-XL-TOPO vector by KpnI and BamHI digestion and ligated into the multiple cloning site of pBSV26*resT* digested with KpnI and BamHI to obtain the plasmid pBSV26*resT*G.

The *bbb26* and *bbb27* genes were FLAG-epitope tagged and individually cloned into the vector pBSV2ex (Guyard *et al*., 2006) under the control of the *flaB* promoter. The *bbb26* and *bbb27* genes were amplified from B31-A genomic DNA and C-terminally FLAG-epitope tagged using Vent DNA polymerase (New England Biolabs) and primer pairs 59 and 61, and 60 and 62, respectively, and cloned into the pCR-XL-TOPO vector. The DNA fragments were removed from the pCR-XL-TOPO vector by digestion with NdeI and KpnI and subcloned individually into pBSV2ex digested with NdeI and KpnI to obtain pBSV2ex-*bbb26*-FLAG and pBSV2ex-*bbb27*-FLAG. These plasmids were transformed into *B. burgdorferi* B31 clone A34. All plasmid constructs were verified by restriction digest and sequence analysis.

### Transformation of *B. burgdorferi*

Transformation of *B. burgdorferi* by electroporation was performed as previously described (Elias *et al*., 2002). Briefly, 10–15 μg of plasmid DNA was resuspended in 8 μl of H_2_O and electroporated into *B. burgdorferi*. Following electroporation, the cells were resuspended in 5 ml of BSK-H complete medium (Sigma) or BSKII medium and allowed to recover for 18–24 h at 35°C. The spirochetes were then plated within solid BSKII medium supplemented with either 200 μg kanamycin ml^−1^, 40 μg gentamicin ml^−1^ or 50 μg spectinomycin ml^−1^.

### Screening of *B. burgdorferi* transformants

*B. burgdorferi* colonies that arose in medium containing antibiotics were inoculated into 20 μl of PCR mixtures with sterile toothpicks. PCR amplification with primers specific for the kanamycin resistance cassette (primers 48 and 49) was used to identify shuttle vector transformants. Allelic exchange transformants were first identified by screening for either the presence of the kanamycin (primers 48 and 49), gentamicin (primers 46 and 47) or streptomycin (primers 55 and 56) resistance cassettes (Table S1). Transformants containing the appropriate resistance cassette were screened for inactivation of targeted genes by PCR with primers described in column 8 of Table 1. The PCR conditions were 94°C for 2 min, followed by 30 cycles of 94°C for 45 s, 55°C for 45 s and 68°C for 3 min in a GeneAmp PCR 9700 thermal cycler (Perkin Elmer) or a DNA engine tetrad 2 thermal cycler (MJ Research). PCR products were separated by agarose gel electrophoresis and visualized by ethidium bromide staining. Colonies of candidate transformants were aspirated with sterile Pasteur pipettes, placed in 5 ml of liquid BSK-H medium (Sigma), or BSKII medium, and allowed to grow to mid to late log phase. Total genomic DNA was then isolated from these cultures with a Wizard genomic DNA purification kit (Promega). PCR performed with total genomic DNA with primers specific for shuttle vectors or cp26 genes was used to further confirm the presence of foreign DNA or the structures of targeted loci in transformants (Table 1, column 8).

### Southern hybridization analysis

Total genomic DNA of *B. burgdorferi* was isolated from 15 ml of cultures with a Wizard genomic DNA purification kit (Promega). Approximately 500 ng of uncut genomic DNA, or 500 ng of genomic DNA digested for 12–20 h with selected restriction enzymes, was separated by gel electrophoresis on a 0.3% agarose gel and visualized by ethidium bromide staining. Genomic DNA was depurinated, denatured and neutralized, and then blotted onto a Biotrans nylon membrane (ICN). A UV stratalinker 1800 (Stratagene) was used to cross-link the DNA to the membrane. The *kan*- and *resT-*specific probes were labeled with ^32^P and hybridizations were performed as previously described (Byram *et al*., 2004).

### Cellular localization

All procedures were performed at 4°C. One litre cultures of *B. burgdorferi* at densities of 5 × 10^7^ spirochetes ml^−1^ were harvested by centrifugation at 10 000 *g* for 10 min. Cell pellets were gently washed two times with 10 mM NaCl in 20 mM HEPES, pH 7.6. Cells were resuspended in 10 ml of 10 mM NaCl in 20 mM HEPES, pH 7.6 and lysed by three passes through a cold French press cell (14 000 lb/in^2^). Unlysed cells and cell debris were removed by centrifugation at 12 000 *g* for 10 min. The soluble and membrane fractions were separated by centrifugation in a Beckman 45Ti rotor (125 000 *g*, 3 h). The membrane pellets were washed in HEPES buffer and harvested by centrifugation in a Beckman 45Ti rotor (125 000 *g*, overnight). The membrane fractions were resuspended in 10 ml of 10 mM NaCl in 20 mM HEPES, pH 7.6. Fractions were normalized according to the total number of spirochetes for immunoblot analysis. Samples were subjected to PAGE with 12% acrylamide. Immunoblots were performed and proteins were detected using monoclonal anti-FLAG (Sigma) (1:500), monoclonal anti-OspB H6831 (Rosa *et al*., 1992) (1:50), monoclonal anti-FlaB H9724 (Barbour *et al*., 1986) (1:200), polyclonal anti-OppAIV (Bono *et al*., 1998) (1:2000) and polyclonal anti-SodA (NIH754) (1:1000) antibodies as previously described (Grimm *et al*., 2004a).

### SodA purification and antibody production

The *sodA* ORF was amplified by PCR from *B. burgdorferi* strain B31-A3 using primers 63 and 64 (Table S1). The resulting DNA fragment was digested with XhoI/PstI and cloned into a similarly digested pBAD/HisA (Invitrogen) vector, generating pBADsodA. SodA-6xHis was overexpressed in *E. coli* Top10 (pLysE) cells (Invitrogen) and purified from the insoluble fraction under denaturing conditions (8 M urea) using a Ni-NTA agarose column (Qiagen) as per manufacturer's instructions. Purified SodA-6xHis was used to raise polyclonal antiserum in a female New Zealand White rabbit at Cocalico Biologicals (Reamstown, PA).

### Proteinase K treatment of *Borrelia* cells

Intact *B. burgdorferi* or *B. burgdorferi* plus 0.1% SDS (Sigma) were treated with proteinase K (Invitrogen) as previously described (Bono *et al*., 1998; Bunikis and Barbour, 1999). Samples were subjected to PAGE with 12% acrylamide and transferred to nitrocellulose membranes. Immunoblots were performed and proteins were detected using monoclonal anti-FLAG, monoclonal anti-OspB, monoclonal anti-FlaB, polyclonal anti-OppAIV antibodies as previously described (Grimm *et al*., 2004a).

## Supplementary material

This material is available as part of the online article from:

http://www.blackwell-synergy.com/doi/abs/10.1111/j.1365-2958.2007.05969.x

(This link will take you to the article abstract).
