# Supplementary material for: Genetic basis for retention of a critical virulence plasmid of Borrelia burgdorferi
Source: Mol Microbiol. 2007 Nov 5;66(4):975–90. doi: 10.1111/j.1365-2958.2007.05969.x (PMC2229028; doi:10.1111/j.1365-2958.2007.05969.x)
Supplement: Supplementary file 1 [file mmi0066-0975-SD1.pdf]

Table S1. Primers used in this study.

Primer	Designation	Sequence 5'-3'
1	BBB29-1100SalIF	GTCGACGCAGATCCCAATACTG
2	resT627-ClaI-RC	CCATCGATTGGAGCAGACTGAGAATCTTACTAAA
3	BBB01-32StuIRC	GAAGGCCTGTTGTTTATACATTATTACCTTCTTTG
4	BBB02-686StuIF	GAAGGCCTGTAAATCGTTCTATTGTCATTAG
5	BBB04RC	ATTCTTTTTCCTGCAAAAGTAGAGCCTTATC
6	BBB07RC	ATTTTCCCAACTACGTTTACAAGACCATTATTTGGG
7	BBB05-3951SalIRC	ACGCGTCGACCATTGCTATTAATTATACCATG
8	BBB06-4788SalIF	ACGCGTCGACCCATATACCCCAATGTAAGTCC
9	BBB08RC	TGCTTTAAGGACATAATAATAAACAGTAATGT
10	BBB09RC	TTCAAATGTGAATTCTCCCTTTTCTCCGTAAGAT
11	BBB08-6054SalIRC	ACGCGTCGACGGGCTATTAATCGGGAGTTTGAAC
12	BBB09-7096SalIF	ACGCGTCGACGGACAATTGGAACGTATCGCACAG
13	BBB13F	AATAGAAATTGTCAAAAGAATTGAGCTTAGTGG
14	BBB16R	TAAATCTTTCCGAAATATTTGGCTCCC
15	BBB14-11014-SalIRC	CGGTCGACGAATTGGATTAGAAGATTTAAGCTTG
16	BBB15-12011-SalIF	CGGTCGACGAATAATTTAAGTGCTTTTAATAACG
17	guaA16159-SalIF	GTCGACGGATGGAATTGTAGGCCG
18	guaA15926-SalIRC	GTCGACGTAAACACTGGATTGTTGCGC
19	BBB22R	AACTAGGCACAGTATTATCATTACTGGAGAG
20	BBB23F	CTATAAAAAGGAAATTCTTGCGGGC
21	BBB22-18435SalIRC	GTCGACGGAAAAATTCCTAATGTCGGC
22	BBB23-20131SalIF	GTCGACCCTTCTAAATTAAAGATTGC

23	BBB23RC	AATATAAAATGCTGCGCCAACAAAAAATCCTGAA
24	BBB28RC	TCACTAGTTTTGTCAGAGTAATCTATGGGC
25	BBB28F	ATTCTGGGTACCTTGTTATTGG
26	BBB28-23564SalIRC	ACGCGTCGACCCATTCTTATTGGTACTGG
27	BBB28-24037SalIF	ACGCGTCGACGAGCCCTTATTACATAGACTTGC
28	BBB22-3-BamF	CGGGATCCGTAGGCTTAAACGGTTTTGGC
29	BBB23-5-BamRC	CGGGATCCCTTCAAACCTTTTTTAGCGTTGGAAG
30	BBB25RKpnI	GGTACCGCATATTCTAAAGTATTTTTTGTATTCTTG
31	BBB28RKpnI	GGTACCTCACTAGTTTTGTCAGAGTAATCTATGGGC
32	BBB24-21361SalIF	ACGCGTCGACGCATATTCTAAAGTATTTTTTGTATTCTTG
33	BBB27F	TGTTTTATGGTTGTTCAACTATATCTTTGG
34	BBB27R	AAAACTTTTTGAGTATATATTCCATCTTTAC
35	BBB25-21821SalIF	ACGCGTCGACCCATTAAAATCAAAGAAAAACAGTATTTCA
36	BBB27-22653SalIRC	GCGTCGACGTCTTGGCGAATGTAAAGATGG
37	BBB26-22579SalIF	ACGCGTCGACGAAGATCTAGAGTAAAAAGCTTTCTC
38	BBB28-23246SalIF	GTCGACGGATGTAAAATGAGTTATTATGTGC
39	BBB26-21923SalIRC	ACGCGTCGACCAAGAGCTGAATTTGTTTTCATTCAT
40	BBB27-23091SalIF	GCGTCGACCCAAAGATATAGTTGAACAACC
41	guaAF	GATTTTGGATCCCAATATAGCC
42	guaAR	CCCATTCTATGGTTGATGGAGGCTTAGA
43	$flgB_{Po}$-XhoI	TAATACTCGAGCTTCAAGGAAGATTT
44	3Gent-ClaI	ATCGATGCGGATCTCGGCTTGAACG
45	KanTerm-Xho	ATCTCGAGCTAGCGCCGTCCCGTCAA
46	flg-PvuII	CAGCTGCCCGAGCTTCAAGGAAGATTTCC
47	Gent-PvuII	CAGCTGGAACGAATTGTTAGGTGGCGG

48	flgPo.Not	GCGGCCGCTACCCGAGCTTCAAGGAAGATT
49	RC.Tkan	GCGCCGTCCCGTCAAGTC
50	pflaB-BamHI 5’	CGCGGATCCTGTCTGTCGCCTCTTGTGGCTTCCGG
51	3GentKpnI	GGTACCGCCGATCTCGGCTTGAACG
52	delta26-27 ex. FWD	TTGGATCATATTGCAAGGTATCC
53	delta26-27 ex. REV	TTCCATTCTTATTGGTACTGG
54	flaB prom-3’+NdeI	GATTGATAATCATATGTCATTCCTCCATG
55	aadA-NdeI 5’	GGAATTCCATATGAGGGAAGCGGTGATCGCCGA
56	aadA-HindIII 3’	AAGCTTTTATTTGCCGACTACCTTGG
57	flaB-XhoI 5’	CCGCTCGAGCTGTCGCCTCTTGTGGCTTC
58	aadA-XhoI 3’	CCGCTCGAGTTATTTGCCGACCTACCTTGG
59	bbb26-NdeI 5’	CATATGAATGAAAACAAATTCAGCTC
60	bbb27-NdeI 5’	CATATGAAGAAGTTTTTAATATCCGT
61	bbb26-KpnI 3’ FLAG	GGTACCTTA<u>CTTGTCGTCATCGTCTTTGTAGTC</u>CTCTAGATCTTCAAATATTT
62	bbb27-KpnI 3’ FLAG	GGTACCTTA<u>CTTGTCGTCATCGTCTTTGTAGTC</u>AGTTAAAAACTTTTTGAGTA
63	sodA-FXh	CATGCTCGAGATGTTTAAGCTGCCAGAACTTGGTTATG
64	sodA-RPs	CCTAGCTGCAGCTAATTAATCACTTCATTGTAAAC
